# Pannexin 1 inhibits rhabdomyosarcoma progression through a mechanism independent of its canonical channel function

**DOI:** 10.1038/s41389-018-0100-4

**Published:** 2018-11-21

**Authors:** Xiao Xiang, Stéphanie Langlois, Marie-Eve St-Pierre, Jessica F. Barré, David Grynspan, Bibianna Purgina, Kyle N. Cowan

**Affiliations:** 1 Molecular Biomedicine Program, Children’s Hospital of Eastern Ontario Research Institute, Ottawa, ON Canada; 2 Department of Cellular and Molecular Medicine, University of Ottawa, Ottawa, ON Canada; 3 Department of Surgery, Children’s Hospital of Eastern Ontario, University of Ottawa, Ottawa, ON Canada; 4 Division of Pathology and Laboratory Medicine, Children’s Hospital of Eastern Ontario, University of Ottawa, Ottawa, ON Canada; 5 Department of Pathology and Laboratory Medicine, The Ottawa Hospital, Ottawa, ON Canada

## Abstract

Rhabdomyosarcoma (RMS) is an aggressive soft tissue sarcoma of childhood thought to arise from impaired differentiation of skeletal muscle progenitors. We have recently identified Pannexin 1 (PANX1) channels as a novel regulator of skeletal myogenesis. In the present study, we determined that PANX1 transcript and protein levels are down-regulated in embryonal (eRMS) and alveolar RMS (aRMS) patient-derived cell lines and primary tumor specimens as compared to differentiated skeletal muscle myoblasts and tissue, respectively. While not sufficient to overcome the inability of RMS to reach terminal differentiation, ectopic expression of PANX1 in eRMS (Rh18) and aRMS (Rh30) cells significantly decreased their proliferative and migratory potential. Furthermore, ectopic PANX1 abolished 3D spheroid formation in eRMS and aRMS cells and induced regression of established spheroids through induction of apoptosis. Notably, PANX1 expression also significantly reduced the growth of human eRMS and aRMS tumor xenografts in vivo. Interestingly, PANX1 does not form active channels when expressed in eRMS (Rh18) and aRMS (Rh30) cells and the addition of PANX1 channel inhibitors did not alter or reverse the PANX1-mediated reduction of cell proliferation and migration. Moreover, expression of channel-defective PANX1 mutants not only disrupted eRMS and aRMS 3D spheroids, but also inhibited in vivo RMS tumor growth. Altogether our findings suggest that PANX1 alleviates RMS malignant properties in vitro and in vivo through a process that is independent of its canonical channel function.

## Introduction

Rhabdomyosarcoma (RMS) is the most common soft tissue sarcoma of childhood^1^. Histopathological classification includes two major subtypes: embryonal (eRMS) and alveolar (aRMS)^2^. eRMS is more frequent, genetically heterogeneous, and associated with a better prognosis^3,4^. On the other hand, aRMS is less common and more aggressive, with a worse outcome^3,4^. RMS cells are positive for myogenic markers and resemble normal muscle progenitors but are unable to complete the multistep process leading to terminal differentiation^5,6^. Despite invasive treatments such as surgery, radiotherapy, and chemotherapy, the prognosis of children with metastatic RMS has not improved and the 5-year survival rate remains <30%^7^, underscoring the need to identify novel therapeutic strategies. Targeting the molecular players involved in the dysregulated myogenic pathways in RMS to promote its differentiation towards skeletal muscle tissue is thought to be a possible new strategy to alleviate RMS malignancy^8^.

Interestingly, we have recently identified Pannexin1 (PANX1) as a novel regulator of myogenic differentiation^9^. PANX1 (known as Panx1 in rodents) levels are very low in undifferentiated human skeletal muscle myoblasts (HSMM), but are up-regulated during their differentiation to promote this process through a mechanism that involves its channel activity^9^. Pannexins are a family of single membrane channel proteins (Panx1, Panx2, and Panx3) that are differentially expressed amongst various cells, tissues, and organs^10^. Panx1 channels at the cell surface act as the major conduit for ATP release^11^ and have been implicated in many physiologic and pathologic processes including calcium wave propagation^12^, vasodilatation^13^, inflammatory responses^14,15^, apoptosis^16–18^, epilepsy^19^, and human immunodeficiency virus infection^20–22^.

Only recently, however, has Panx1 been studied in the context of cancer. Initial reports showed that Panx1 levels are low in glioma cell lines and that Panx1 over-expression suppresses rat C6 glioma tumor formation^23^. It was then reported that Panx1 levels are up-regulated in murine melanoma cell lines and correlated with their aggressiveness^24^. Loss of Panx1 attenuated melanoma progression through reversion to a melanocytic phenotype^24^. In human cancer, PANX1 levels were shown to be down-regulated in keratinocyte tumors^25^. On the other hand, high *PANX1* mRNA expression is correlated with poor overall survival in breast cancer patients^26^. Furthermore, a mutation encoding a truncated form of PANX1 is recurrently enriched in highly metastatic breast cancer cells^27^. This truncated version permits metastatic cell survival in the vasculature by enhancing PANX1 channel activity. Importantly, PANX1 channel blockade reduced breast cancer metastasis efficiency in vivo^27^. Altogether these studies indicate that Panx1/PANX1 expression and/or channel activity are altered in some forms of cancer, may be correlated with their aggressiveness, and that restoration of its levels and/or activity alleviate tumor malignant characteristics. Here, we show that PANX1 is down-regulated in human eRMS and aRMS primary tumor specimens and patient-derived cell lines, when compared to normal differentiated skeletal muscle cells and tissue. Once expressed in eRMS (Rh18) and aRMS (Rh30) cells, PANX1 did not overcome the inability of RMS to reach terminal differentiation but rather significantly decreased their malignant properties in vitro and in vivo. Based on the current knowledge of PANX1 channels, our data obtained from dye uptake assays, utilization of PANX1 channel inhibitors, and expression of PANX1 mutants deficient in channel activity, altogether indicate that PANX1 tumor suppressive roles in RMS do not require its canonical channel activity suggesting the existence of novel PANX1 functions.

## Results

### PANX1 is down-regulated in RMS

**Fig. 1:**
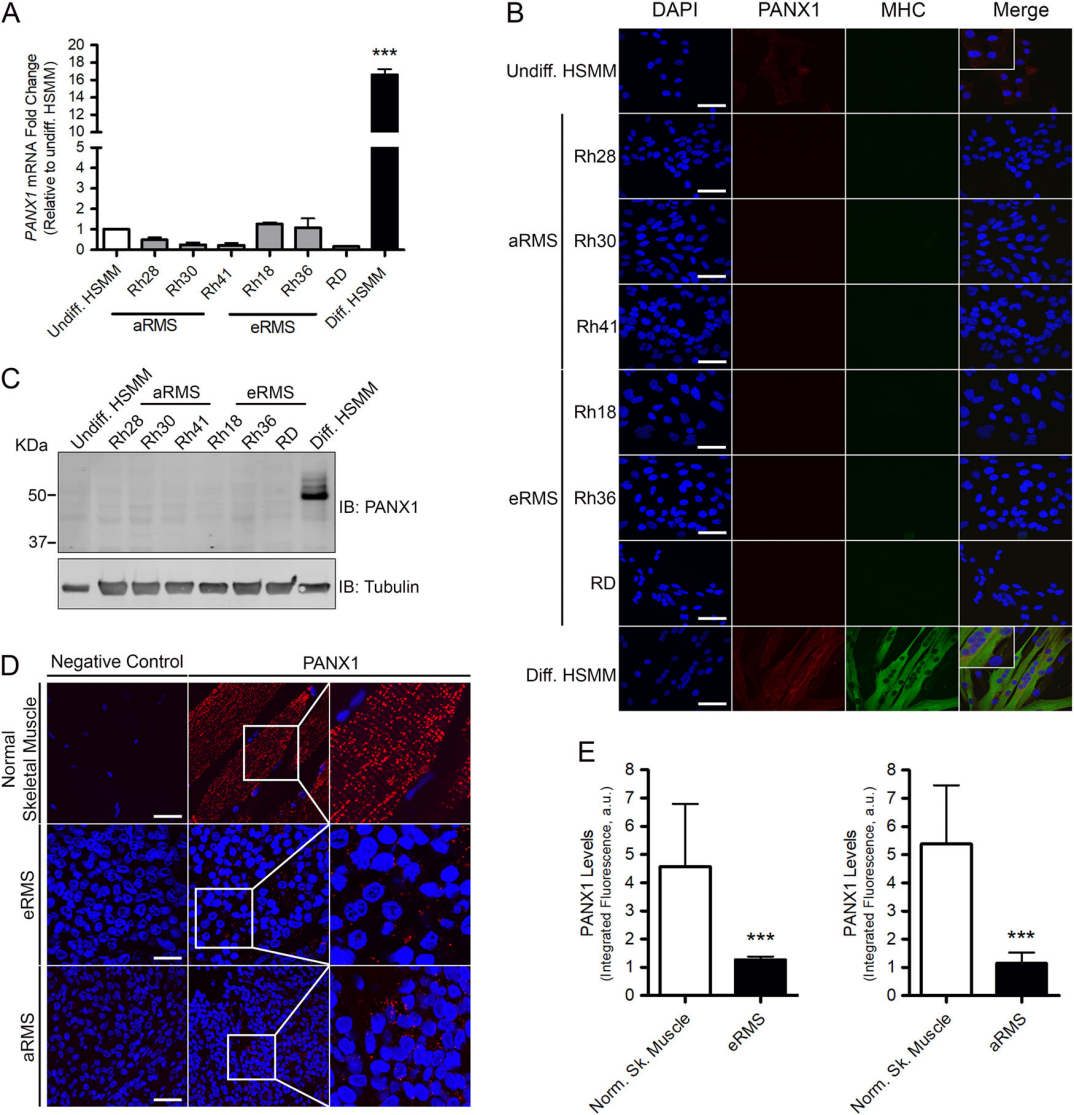
PANX1 is down-regulated in human eRMS and aRMS. **a** RT-qPCR analysis, **b** immunofluorescent labeling (PANX1, red), and **c** Western blot of six patient-derived RMS cell lines (three aRMS and three eRMS), undifferentiated (Undiff.) and differentiated (Diff.) human skeletal muscle myoblasts (HSMM) revealed that PANX1 transcript and protein levels are down-regulated when compared to differentiated HSMM. Myosin heavy chain (MHC, green) was used as a marker for myogenic differentiation. Tubulin was used as a loading control. ***P < 0.001 compared to differentiated HSMM. Results are expressed as mean ± s.d. **d** Representative images of human RMS primary tumors and normal skeletal muscle from formalin-fixed, paraffin-embedded sections were immunolabeled for PANX1 (red), which was quantified in **e**. The negative control, without primary antibodies, confirmed labeling specificity. ***P < 0.001 compared to normal skeletal muscle. Results are expressed as mean ± s.d. Blue = nuclei, bars = 30 µm

Quantitative real-time PCR, immunofluorescence microscopy, and Western blotting were performed to examine PANX1 expression in a panel of patient-derived aRMS (Rh28, Rh30, Rh41) and eRMS (Rh18, Rh36, RD) cell lines compared to those of undifferentiated and differentiated HSMM. *PANX1* expression was significantly increased in differentiated HSMM compared to undifferentiated cells (Fig. 1a). *PANX1* transcript levels were low in all RMS cell lines tested and were comparable to that of undifferentiated HSMM (Fig. 1a). In keeping with these data, immunolabeling (Fig. 1b) and Western blot (Fig. 1c) analysis revealed that PANX1 is highly expressed in differentiated HSMM, while PANX1 levels are very low or below detectable levels in all RMS cell lines, as well as in undifferentiated HSMM.

To confirm the pertinence of these findings, we examined PANX1 levels in RMS tumor specimens. PANX1 was immunolabeled in 13 pediatric RMS tumors (seven eRMS and six aRMS), as well as in seven pediatric normal skeletal muscle samples. As expected^9^, PANX1 was detected as a punctate labeling in normal muscle tissue (Fig. 1d). While PANX1 was also detected as punctate structures in RMS tumors (Fig. 1d), its levels were strikingly lower in both RMS subtypes compared to normal skeletal muscle tissue (Fig. 1e). Altogether these results indicate that PANX1 expression is down-regulated in aRMS and eRMS.

### PANX1 expression impedes RMS cell proliferation and migration

**Fig. 2:**
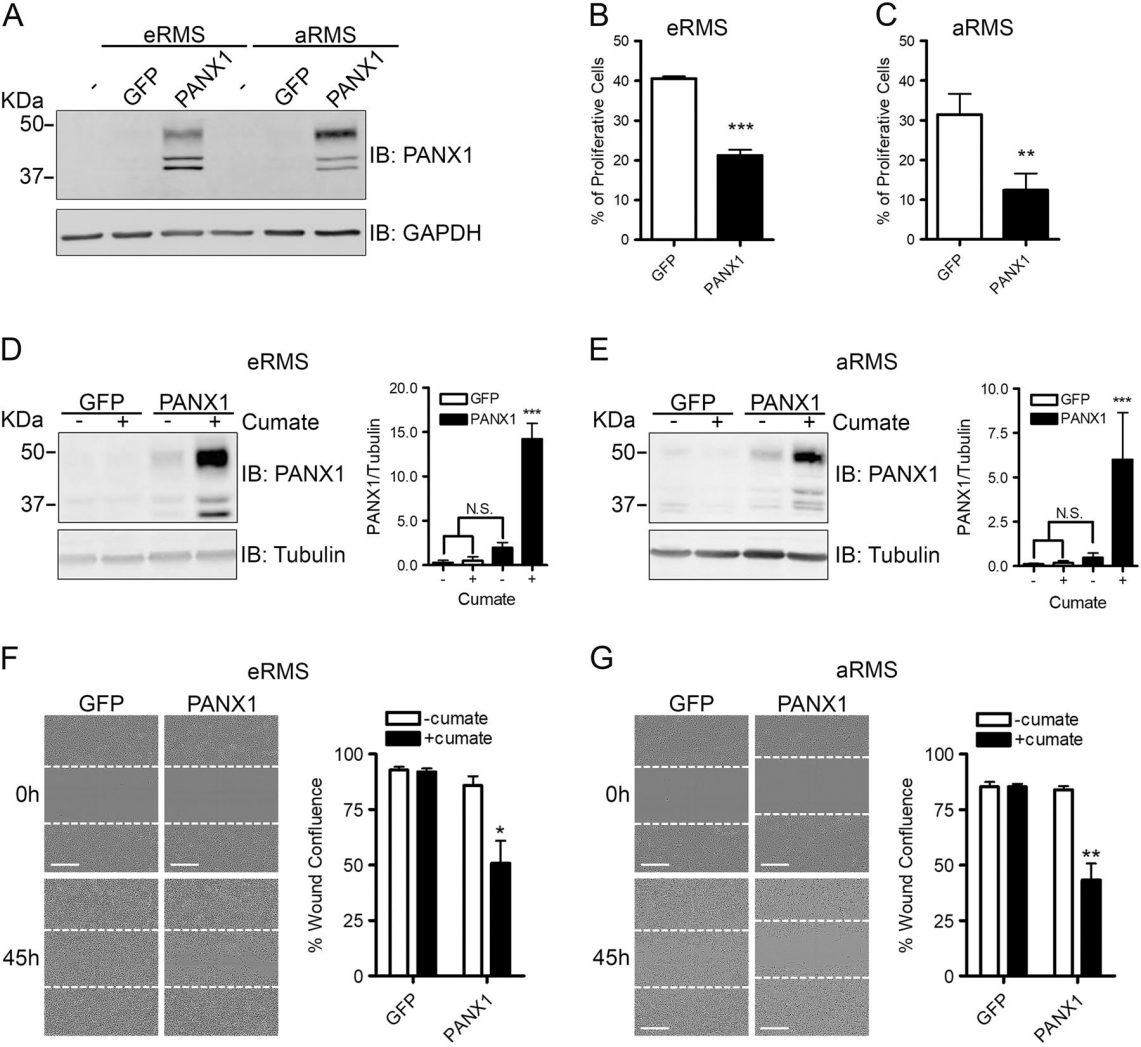
PANX1 expression inhibits RMS cell proliferation and migration. eRMS (Rh18) and aRMS (Rh30) cells ectopically expressing PANX1 were analyzed by BrdU incorporation and scratch wound migration assays. **a** Representative Western blot of eRMS (Rh18) and aRMS (Rh30) cells transiently transfected with PANX1. GAPDH was used as a loading control. BrdU incorporation assay showed a significant reduction of proliferation in eRMS (Rh18) (**b**) and aRMS (Rh30) (**c**) cells over-expressing PANX1. ***P < 0.001 and **P < 0.01 compared to GFP. Western blot analysis and quantification of eRMS (Rh18) (**d**) and aRMS (Rh30) (**e**) inducible stable cells over-expressing PANX1 after treatment with 30 µg/mL cumate for 24 h. Tubulin was used as loading control. ***P < 0.001 compared to GFP without cumate, GFP with cumate, and PANX1 without cumate. N.S.: not significant. Representative pictures and quantification of stable eRMS (Rh18) (**f**) and aRMS (Rh30) (**g**) cells, treated with or without cumate to induce PANX1 over-expression, subjected to scratch wound assay for 45 h. The dotted lines show cell boundaries after initial scratch. The confluence of the wound areas was quantified 45 h post wounding, which showed a significant reduction in cumate-treated PANX1 over-expressing stable eRMS (Rh18) (**f**) and aRMS (Rh30) (**g**) compared to their respective controls. **P < 0.01, *P < 0.05 compared to GFP without cumate, GFP with cumate, and PANX1 without cumate. Results are expressed as mean ± s.d. Bars = 300 µm

Next, we wanted to assess whether restoration of PANX1 expression can reduce RMS malignant properties. A representative cell line for each RMS subtype, Rh18 for eRMS and Rh30 for aRMS, have been used. PANX1 was detected as multiple bands (~38–50 kDa) by Western blot (Fig. 2a) reflecting its various glycosylation degrees^9,28–30^. eRMS and aRMS cells expressing PANX1 were submitted to a BrdU incorporation assay along with their respective control GFP-expressing cells. PANX1 expression reduced the proliferation of eRMS (Fig. 2b) and aRMS (Fig. 2c) cells by ~50–60%. Based on these promising results, we generated stable Rh18 and Rh30 cell lines using a cumate-inducible gene expression system to regulate PANX1 expression. These cell lines show a significant induction of PANX1 levels only in cumate-treated PANX1-transductants (Fig. 2d, e). Using these stable cell lines, a scratch wound migration assay showed a reduction of migration by PANX1-expressing eRMS (Fig. 2f) and aRMS (Fig. 2g) cells. Collectively, these results reveal that introduction of PANX1 in RMS reduces their growth and migratory potential in vitro.

### PANX1 expression does not trigger RMS terminal differentiation

**Fig. 3:**
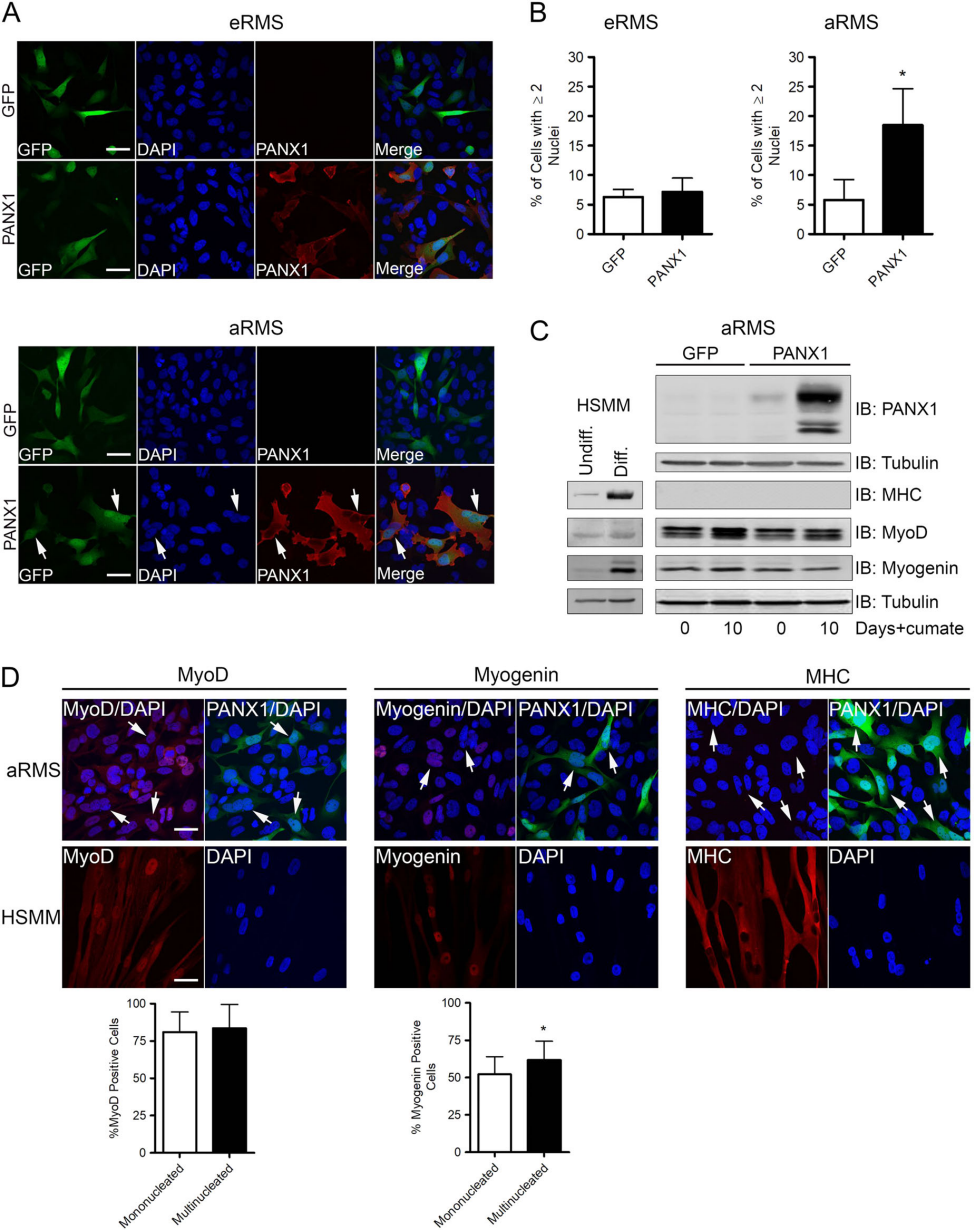
PANX1 expression does not trigger RMS cell terminal differentiation. **a** PANX1 (red) immunofluorescence labeling of eRMS (Rh18) and aRMS (Rh30) transfected with PANX1 or the control vector GFP. While no morphological changes were observed in eRMS cells, a population of PANX1 over-expressing aRMS cells was multinucleated (arrows). Blue = nuclei, bars = 20 µm. **b** Quantification of multinucleation (% of cells with ≥2 nuclei) of aRMS (Rh30) cells showed a significant increase with PANX1 over-expression. *P < 0.05 compared to GFP. **c** Cumate-inducible stable aRMS (Rh30) cells were cultured in growth media with 30 µg/mL cumate for 10 days, and analyzed for MHC, MyoD, and myogenin levels. Cells were collected for analysis immediately prior to cumate treatment on Day 0. Undifferentiated and differentiated HSMM were used as myogenic marker controls. Tubulin was used as a loading control. **d** Representative immunofluorescence images of aRMS (Rh30) cells transfected with PANX1 and labeled with MyoD, myogenin, or MHC (red). Cells transfected with PANX1 (GFP-positive) are shown in green. Differentiated HSMM were used as positive controls. The percentage of mononucleated and multinucleated (arrows) PANX1-expressing cells positive for MyoD, myogenin, and MHC were quantified in a double-blinded manner. *P < 0.05 compared to mononucleated cells. Bars = 20 µm. Results are expressed as mean ± s.d

Having previously shown that PANX1 expression promotes HSMM fusion and differentiation^9^, we wanted to examine whether PANX1 could induce RMS differentiation. First, eRMS (Rh18) and RMS (Rh30) cells were transfected with GFP or PANX1, immunolabeled for PANX1, and nuclei were counted in transfected cells. About 20% of aRMS cells expressing PANX1 contained two or more nuclei (Fig. 3a, arrows; Fig. 3b, quantification) while almost all control cells were mononucleated. This was not observed in eRMS cells, as 7–8% of both control and PANX1-expressing cells were multinucleated (Fig. 3a, b). In order to determine whether the change in phenotype observed in PANX1-expressing aRMS cells was associated with myogenic differentiation, the levels of several myogenic factors were examined over a period of 10 days. MyoD and myogenin levels were relatively unchanged in both control and PANX1-expressing aRMS cells while myosin heavy chain (MHC) remained below detectable limits (Fig. 3c). As multinucleated cells constituted only a small proportion of the cells culture, myogenic factor expression was then specifically examined in mononucleated and multinucleated cells by immunofluorescence (Fig. 3d). While the proportion of MyoD-positive cells remained unchanged despite the nucleation status of PANX1-expressing cells, more multinucleated PANX1-expressing aRMS cells were myogenin-positive compared to their mononucleated counterparts. MHC was again not detected in PANX1-expressing cells indicating that terminal differentiation had not been reached. These results suggest that although an early fusion stage of myogenesis may have been attained in aRMS cells, PANX1 expression was not sufficient to trigger their differentiation.

### PANX1 expression prevents 3D RMS spheroid formation and induces their regression by apoptosis

**Fig. 4:**
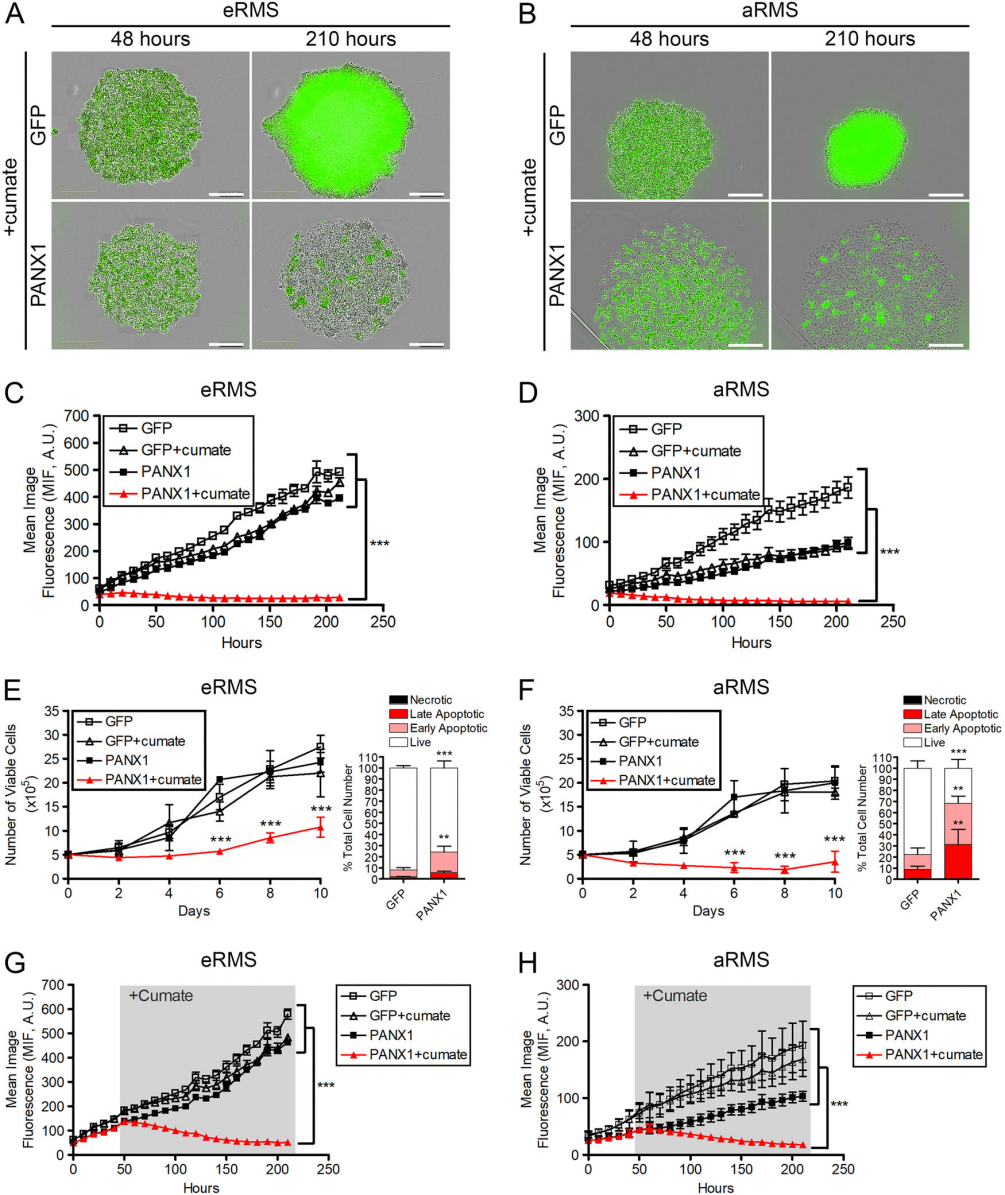
PANX1 expression blocked formation and induced regression of 3D RMS spheroids through induction of cell apoptosis. Stable eRMS (Rh18) and aRMS (Rh30) cells were pre-treated and maintained with 30 µg/mL cumate to induce PANX1 expression and 3D spheroid formation was monitored over 210 h. Representative images taken at 48 and 210 h clearly show that over-expression of PANX1 prevented eRMS (Rh18) (**a**) and aRMS (Rh30) (**b**) spheroid formation. Mean image fluorescence (MIF), a surrogate measurement for spheroid size, was measured over time for both eRMS (Rh18) (**c**) and aRMS (Rh30) (**d**) cells. A.U. arbitrary units. Bars = 1000 µm. The viability of stable eRMS (Rh18) (**e**; left panel) and aRMS (Rh30) (**f**; left panel) cells grown in suspension on agar for 10 days in the absence or presence of cumate was assessed every 2 days. Viable cell counts showed a significantly reduced number of viable cells within cumate-induced PANX1 RMS spheroids. ***P < 0.001 compared to untreated GFP cells, GFP cells treated with cumate, and untreated PANX1 cells. After being grown in suspension for 4 days, live (Annexin V−, 7-AAD−), early apoptotic (Annexin V+, 7-AAD-), late apoptotic (Annexin V+, 7-AAD+) and necrotic (Annexin V−, 7-AAD+) cell populations were quantified as percentages of the total cell population and revealed a significant increase in apoptotic cell populations within PANX1 over-expressing eRMS (Rh18) (**e**; right panel) and aRMS (Rh30) (**f**; right panel) spheroids. ***P < 0.001, **P < 0.01 compared to respective control cells expressing GFP. Results are expressed as mean ± s.d. In the spheroid regression assay, stable eRMS (Rh18) and aRMS (Rh30) cells were first allowed to form spheroids for 48 h before being treated with cumate to induce PANX1 expression. Spheroid growth was monitored for a total of 210 h. While the mean image fluorescence (MIF) of control stable eRMS (Rh18) (**g**) and aRMS (Rh30) (**h**) spheroids progressively increased over time, a continuous and significant decrease of MIF was measured upon PANX1 expression. ***P < 0.001 compared to untreated GFP cells, GFP cells treated with cumate, and untreated PANX1 cells. Results are expressed as mean ± s.d. A.U. arbitrary units. Bars = 1000 µm

To examine the role of PANX1 in tumor formation, we first utilized 3D spheroid cultures as this model more closely resembles in vivo tumors^31^. Aggregation and compaction in GFP control and PANX1-expressing eRMS (Rh18) and aRMS (Rh30) cells were first observed 48 h post seeding (Fig. 4a, b). While the growth of control spheroids continued to increase over time, PANX1 expression prevented the formation of eRMS (Fig. 4c) and aRMS (Fig. 4d) spheroids. Only loose aggregates were formed by PANX1-expressing cells, which gradually lost their constitutive GFP fluorescence possibly due to cell death.

To test this possibility, the viability of eRMS and aRMS spheroids was determined over a period of 10 days. As expected, control cells showed a continuous increase in viable cell number. By contrast, PANX1-expressing eRMS (Fig. 4e, left panel) and aRMS (Fig. 4f, left panel) cells displayed a significant reduction in viability compared to control cells with the overall number of live PANX1-expressing aRMS cells remaining unchanged for 10 days. To examine whether these effects were due to apoptosis, flow cytometry was performed using Annexin V and 7-AAD^32^. In order to capture early apoptotic events, cells were examined after being in suspension for 4 days. The percentage of early apoptotic cells was significantly higher amongst PANX1-expressing eRMS cells compared to control cells (Fig. 4e, right panel), while both early and late apoptotic populations were increased in PANX1-expressing aRMS cells (Fig. 4f, right panel).

Of particular relevance in terms of therapeutic intervention for RMS treatment, we next examined whether PANX1 was able to trigger RMS spheroid regression. As Rh18 (eRMS) and Rh30 (aRMS) cells formed sizable spheroids 48 h after being in suspension, cumate treatment was initiated at that time point to induce PANX1 expression. While control spheroids displayed an ongoing growth, there was a continuous decrease of fluorescence in PANX1-expressing eRMS and aRMS spheroids after initiation of cumate treatment eventually reaching a level that was similar to that of cells in suspension prior to spheroid formation (Fig. 4g, h). Taken together, our data demonstrate that PANX1 expression prevents RMS spheroid formation and triggers their regression through the induction of apoptosis.

### PANX1 expression decreases RMS tumor growth in vivo

**Fig. 5:**
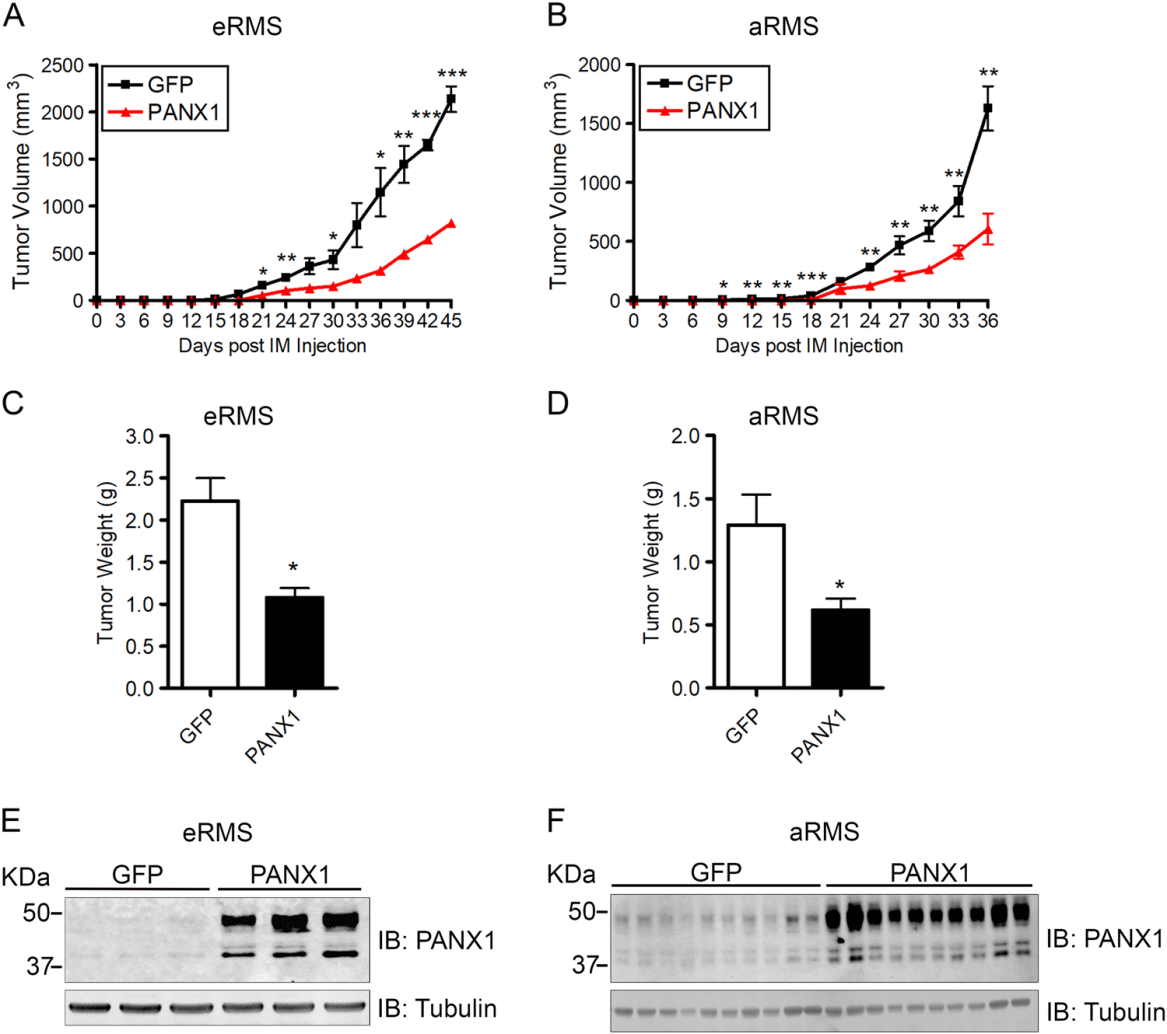
Expression of PANX1 decreases RMS tumor growth in vivo. Stable GFP control and PANX1 over-expressing eRMS (Rh18) and aRMS (Rh30) cells were injected orthotopically into the left and right gastrocnemius of the mice (mice randomly assigned, not a blinded method). PANX1 expression was maintained by intraperitoneal injection of cumate every 3 days. PANX1 over-expression in eRMS (Rh18) (**a**) and aRMS (Rh30) (**b**) cells led to a significantly reduced growth rate compared to their respective GFP controls. At endpoint, PANX1-expressing eRMS (Rh18) (**c**) and aRMS (Rh30) (**d**) xenografts weighted significantly less than the control tumors. Day 0 denotes the day of cell intramuscular (IM) injection. *P < 0.05, **P < 0.01, ***P < 0.001 compared to GFP. Results are expressed as mean ± s.d. Western blots of eRMS (Rh18) (**e**) and aRMS (Rh30) (**f**) xenografts demonstrate successful induction of PANX1 by cumate in vivo. Tubulin was used as loading control

The numerous tumor-suppressive effects of PANX1 in RMS cells in vitro prompted us to explore its impact on tumor growth in vivo. Cumate-treated eRMS (Rh18) and aRMS (Rh30) cells expressing the empty vector (GFP) or PANX1 were injected into the gastrocnemius muscle of mice and kept under cumate treatment. Control eRMS and aRMS cells formed rapidly growing tumors reaching the ethical endpoint of ∼2000 mm^3^ after 45 days and 36 days, respectively (Fig. 5a, b). Conversely, tumors formed by PANX1-expressing eRMS and aRMS cells grew significantly slower (Fig. 5a, b), which was clearly depicted by a reduction of ~50% in their tumor weight at the endpoint (Fig. 5c, d). Induction of PANX1 expression in all eRMS and aRMS xenografts was confirmed by Western blotting (Fig. 5e, f). Overall, these results revealed that PANX1 expression reduces RMS tumor growth in vivo.

### PANX1-mediated tumor suppressive properties are not abrogated by PANX1 channel inhibition

**Fig. 6:**
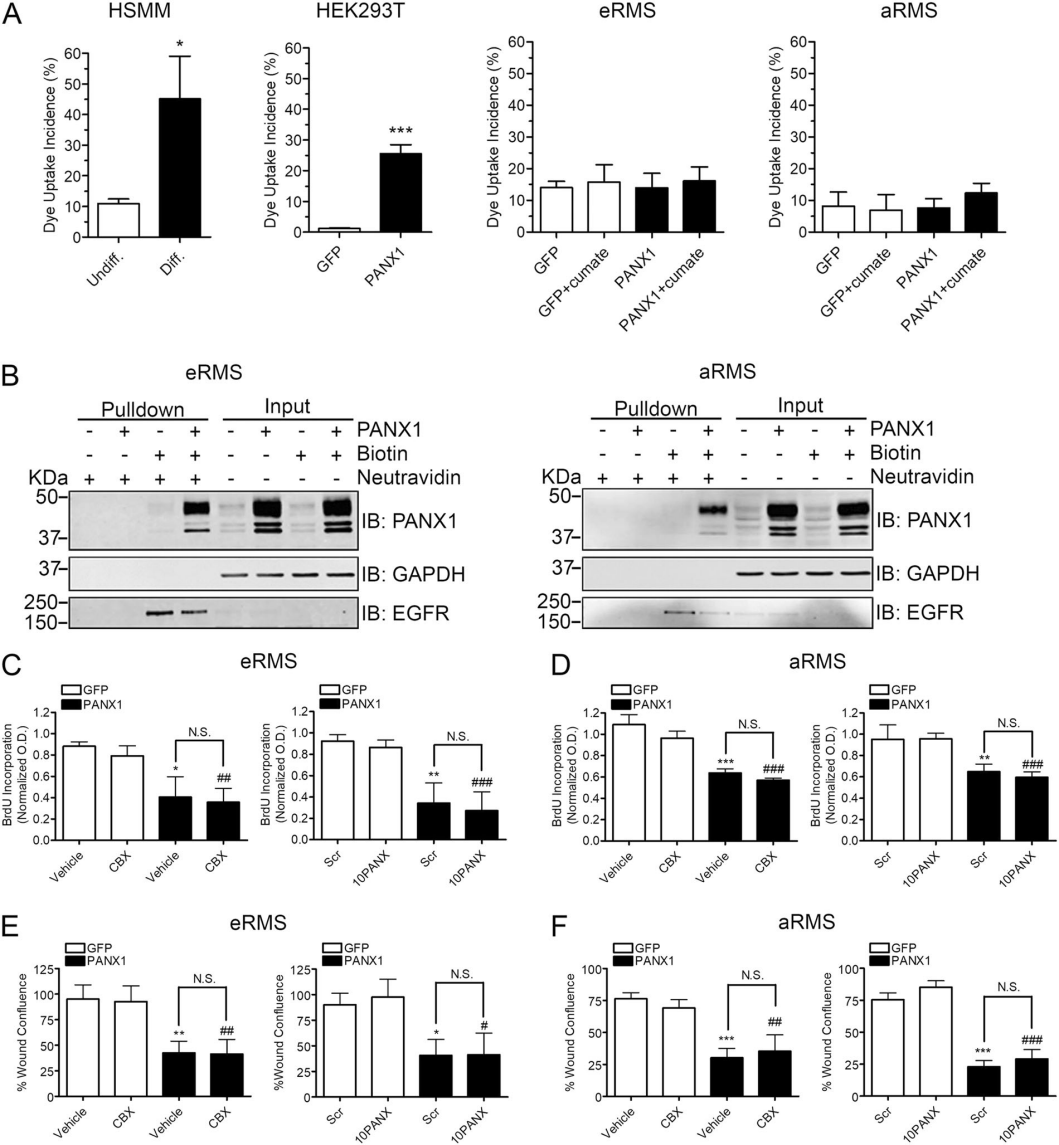
PANX1 is incapable of dye uptake in Rh18 and Rh30 cells and its tumor suppressive functions are not reduced by PANX1 channel inhibitors. **a** PANX1 expression did not augment dye uptake incidence in eRMS (Rh18) and aRMS (Rh30) cells following mechanical stimulation. By contrast, differentiated (Diff.) HSMM, which express high endogenous levels of PANX1 displayed a significant increase of dye uptake, as compared to undifferentiated (Undiff.) HSMM, which express low PANX1 levels. HEK293T cells were used as a positive control for ectopic PANX1 expression using the same construct that was utilized to engineer the stable eRMS (Rh18) and aRMS (Rh30) cell lines. HSMM: *P < 0.05 compared to Undiff.; HEK293T: ***P < 0.001 compared to GFP. Results are expressed as mean ± s.d. **b** Western blot analysis of cell surface biotinylation assays showing localization of PANX1 in the plasma membrane fraction of both eRMS (Rh18) and aRMS (Rh30) cells. GAPDH was used as a marker for cytosolic proteins, while EGFR (epidermal growth factor receptor) was used as a marker for plasma membrane proteins. eRMS (Rh18) and aRMS (Rh30) stable cells expressing PANX1 or control GFP vector were incubated for 48 h with either CBX (100 µM) or 10PANX (200 µM) and subjected to BrdU assays. As shown earlier, PANX1 expression resulted in a significant reduction of eRMS (**c**) and aRMS (**d**) cell proliferation, which remained unchanged when treated by CBX and 10PANX compared to the vehicle DMSO control or the scramble (Scr) peptide. Normalized optical density (OD) was calculated compared to that of the respective untreated GFP control cells. eRMS (Rh18) and aRMS (Rh30) stable cells expressing PANX1 or control GFP vector were subjected to a scratch wound assay in the presence or absence of CBX (100 µM) and 10PANX (200 µM). The percent wound confluence was calculated 45 h post-scratch using the IncuCyte Analysis System. The reduction of wound confluence induced by PANX1 expression was not altered by CBX nor 10PANX compared to their respective controls in both eRMS (Rh18) (**e**) and aRMS (Rh30) (**f**) cells. *P < 0.05, **P < 0.01, and ***P < 0.001 compared to GFP vehicle or GFP Scr controls. ##*P* < 0.01 and ###*P* < 0.001 compared to CBX- or 10PANX-treated GFP controls. N.S.: not significant. Results are expressed as mean ± s.d

In order to understand the mechanism by which PANX1 reduces RMS malignant properties, we assessed its channel function using dye uptake. As expected, differentiated HSMM, which express high PANX1 levels, showed a significant increase in dye uptake incidence compared to undifferentiated HSMM, which express only low levels of PANX1 (Fig. 6a). Similar to undifferentiated HSMM, eRMS and aRMS cells showed low dye uptake incidence. Surprisingly, PANX1 expression did not induce dye uptake in eRMS nor aRMS cells (Fig. 6a). As a control, the PANX1 construct used to generate the stable RMS cell lines was transfected into HEK293T cells, which have been utilized in many studies examining Panx1 channel activity^16,29,30^, and showed a significant elevation of dye uptake compared to GFP-expressing cells (Fig. 6a). In order to eliminate the possibility that the lack of dye uptake by PANX1 in RMS cells was due to an absence of its localization at the plasma membrane, cell surface biotinylation assays were performed. Ectopic PANX1 was found at the cell surface of both eRMS and aRMS cells (Fig. 6b). Based on these findings, we hypothesized that the PANX1 tumor suppressive effects are independent of its channel function. eRMS and aRMS cells were thus subjected to a BrdU incorporation assay in the presence or absence of the PANX1 channel blocker carbenoxolone (CBX) and the specific mimetic peptide inhibitor of PANX1, 10PANX. Similar to our previous findings, the vehicle-treated or scramble (Scr) peptide-treated PANX1-expressing eRMS (Fig. 6c) and aRMS (Fig. 6d) exhibited significantly reduced cell proliferation compared to their respective control cells. However, this PANX1-mediated effect was not altered by CBX or 10PANX (Fig. 6c, d). Similarly, the significant reduction of cell migration mediated by PANX1 was not affected or reversed by the inhibitors in both eRMS (Fig. 6e) and aRMS (Fig. 6f) cells. Taken together, these data suggest that the PANX1-mediated tumor suppressive effects in RMS are independent from its canonical channel activity.

### Characterization of channel deficient PANX1 mutants

**Fig. 7:**
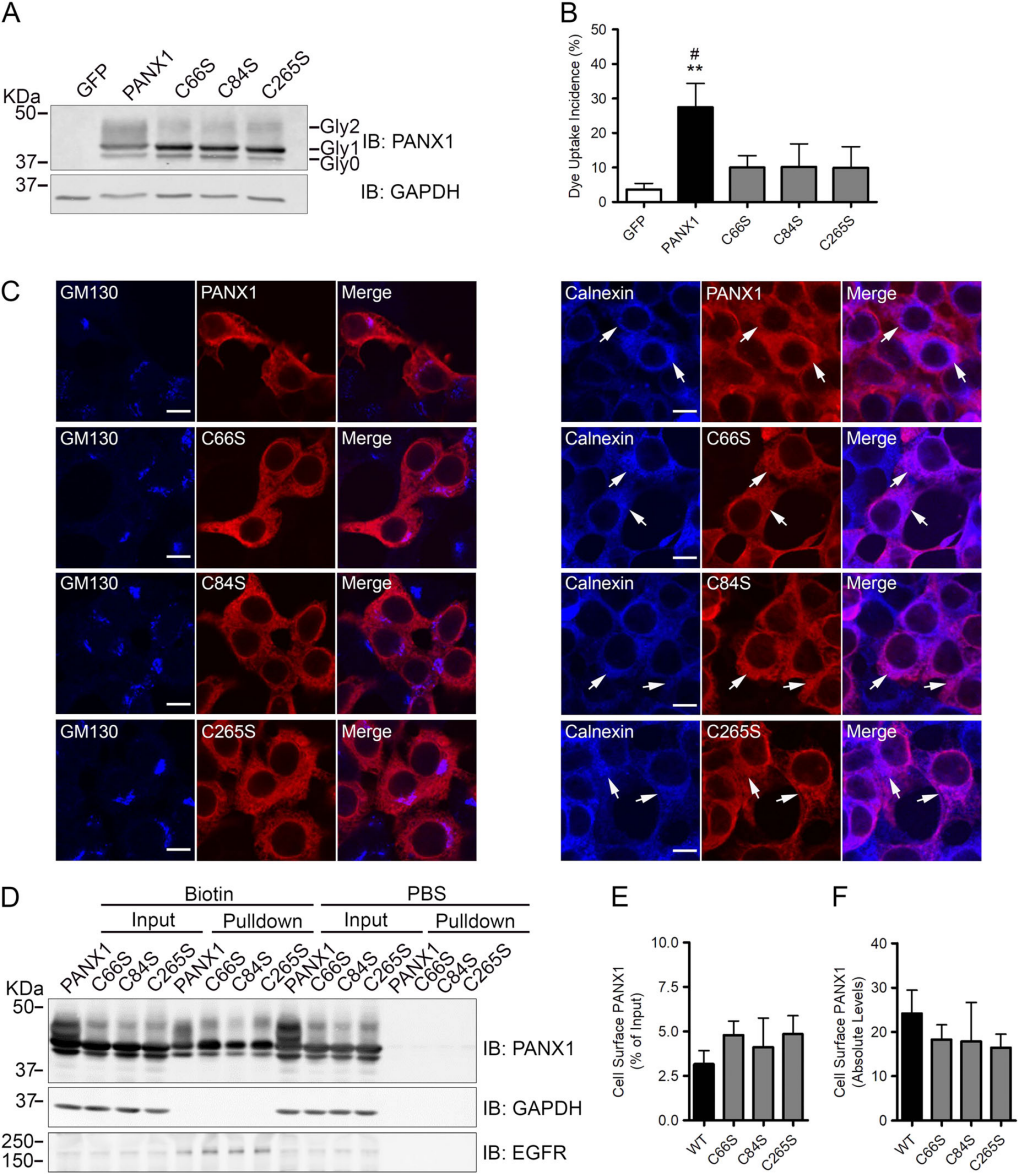
C66S, C84S, and C265S PANX1 mutants are channel deficient. **a** Western blot of C66S, C84S, and C265S PANX1 mutants show banding patterns similar to wild-type PANX1 in HEK293T cells. The Gly0, Gly1, and Gly2 species of PANX1 are indicated. GAPDH was used as a loading control. **b** Expression of C66S, C84S, and C265S mutants in HEK293T cells did not increase dye uptake incidence, unlike wild-type PANX1 expressing cells. **P < 0.001 compared to GFP, #*P* < 0.01 compared to C66S, C84S, and C265S. **c** HEK293T cells were transfected with PANX1, C66S, C84S, and C265S constructs. The cells were then co-labeled for PANX1 (red) together with GM130 (Golgi apparatus marker) or calnexin (ER marker) in blue. Representative images show some co-localization (arrows) of PANX1 and the three mutants with calnexin. Bars = 8 µm. **d** Cell surface biotinylation experiments demonstrate that PANX1, as well as all three PANX1 mutants, are detected at the plasma membrane of HEK293T cells. GAPDH was used as a marker for cytosolic proteins, while EGFR was used as a marker for plasma membrane proteins. Densitometric analysis and quantification of cell surface biotinylation experiments show that all three mutants are localized at the cell surface in the same amount as PANX1. Cell surface expression was calculated relative to the total protein in input lanes (**e**), and was also calculated as absolute protein levels in the pulldown lanes (**f**)

In order to confirm these data, we sought to generate PANX1 mutants that constitutively lack channel activity. Bunse and colleagues showed that substitution of a single cysteine residue with serine at amino acid positions 66, 84, and 264 resulted in complete loss of Panx1 channel function^33^. We thus mutated the corresponding cysteine residues at position 66, 84, and 265 of human PANX1 to generate the C66S, C84S, and C265S mutants, respectively. When expressed in HEK293T cells, similar to PANX1, all three mutants exhibit multiple bands by Western blot (Fig. 7a) known as the core unglycosylated protein (Gly0), a high mannose-glycosylated species associated with the ER (Gly1), and the extensively glycosylated species (Gly2) that is modified in the Golgi apparatus and then traffics to the plasma membrane^28–30^. The C66S, C84S, and C265S mutants displayed a slight enrichment in the Gly1 species expression when compared to PANX1. As opposed to wild-type PANX1, all three mutants exhibited dramatically reduced dye uptake incidence following mechanical stimulation when expressed in HEK293T cells (Fig. 7b). Similar results were obtained when high (50 mM) potassium medium was used to activate PANX1 channels (data not shown)^15^. In order to examine their localization, PANX1 and the C66S, C84S, and C265S mutants were co-labeled with Golgi apparatus and endoplasmic reticulum (ER) markers in HEK293T cells. As shown in Fig. 7c, there was no evident co-localization of PANX1, or the PANX1 mutants, with the Golgi apparatus marker GM130. However, a proportion of the intracellular pool of both PANX1 and the PANX1 mutants was found co-localized with the ER marker calnexin (Fig. 7c; arrows). In order to quantify PANX1 mutants at the cell surface of HEK293T cells and eliminate the possibility that the lack of dye uptake by PANX1 mutants was due to reduced or absence of localization at the plasma membrane, cell surface biotinylation assays were performed. As shown in Fig. 7d–f, ectopic PANX1 and all three PANX1 mutants were found at the plasma membrane as ~3–5% of their total expression was detected at the cell surface (Fig. 7e). Their absolute levels at the plasma membrane were also similar (Fig. 7f). Together, our results suggest that similar to PANX1, all three PANX1 mutants are localized in the ER and at the cell surface of HEK293T cells. However, unlike PANX1, the C66S, C84S, and C265S mutants exhibit deficient channel function.

### PANX1 mutants reduce RMS malignant properties despite being deficient in channel function

**Fig. 8:**
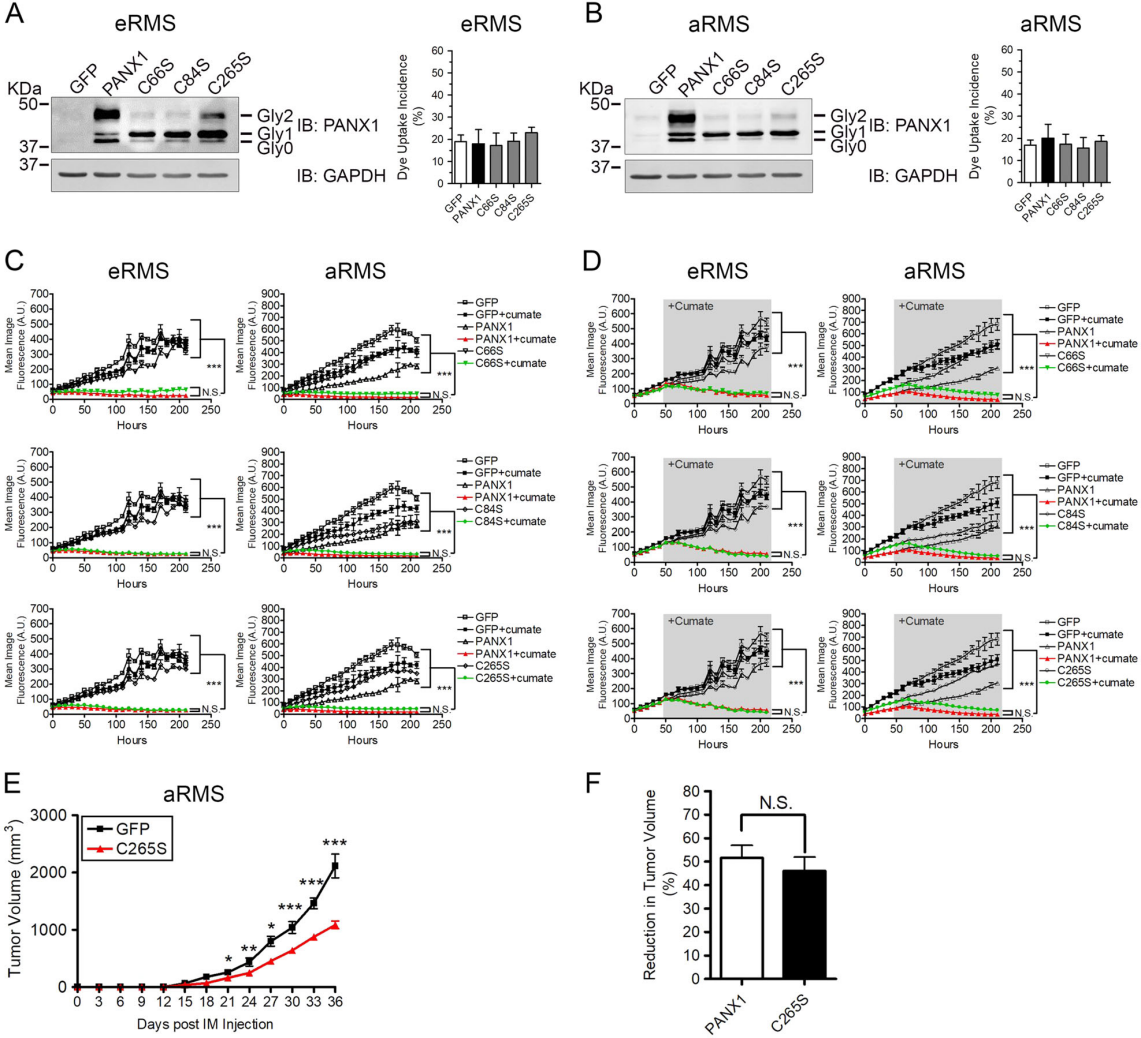
Channel defective PANX1 mutants reduce RMS tumor growth. Western blot of C66S, C84S, and C265S PANX1 mutants compared to wild-type PANX1 in eRMS (Rh18) (**a**) and aRMS (Rh30) cells (**b**). The Gly0, Gly1, and Gly2 species of PANX1 are indicated. GAPDH was used as a loading control. Similar to wild-type PANX1 expressing cells, no increase in dye uptake was observed when C66S, C84S, and C265S mutants were expressed in eRMS (Rh18) (**a**) and aRMS (Rh30) cells (**b**). 3D spheroid formation (**c**) and regression (**d**) assays described previously were performed using inducible eRMS (Rh18) and aRMS (Rh30) stable cell lines expressing either the C66S, C84S, or C265S PANX1 mutant and compared to cells expressing wild-type PANX1, as well as to GFP control cells. In both eRMS and aRMS cells, expression of PANX1 mutants inhibited formation of 3D spheroids (**c**) and caused their regression (**d**) similar to wild-type PANX1. ***P < 0.001 compared GFP; GFP treated with cumate; PANX1; and C66S or C84S or C265S mutant. N.S.: not significant. aRMS (Rh30) cells over-expressing the C265S mutant or the GFP control vector were injected orthotopically into the left and right gastrocnemius of the mice, respectively. Intraperitoneal injection of cumate was performed every 3 days. Expression of the C265S mutant significantly reduced aRMS xenograft growth rate (**e**). Expression of PANX1 and the C265S mutant both resulted in a diminution of ~50% in tumor volume when compared to control xenografts (**f**). N.S.: not significant. Day 0 denotes the day of intramuscular (IM) cell injection. *p < 0.05, **p < 0.01, ***p < 0.001 compared to GFP. Results are expressed as mean ± s.d

After having established that the C66S, C84S, and C265S PANX1 mutants are deficient in channel function, we next wanted to utilize these constructs to assess their potential at alleviating RMS malignant properties. When expressed in eRMS (Fig. 8a) and aRMS (Fig. 8b) cells, all three mutants were similar to the wild-type protein as the Gly0, Gly1, and Gly2 species could be detected. However, Gly1 was the main species expressed for the PANX1 mutants likely reflecting intracellular localization in the ER. Similar to wild-type PANX1, all three mutants were incapable of dye uptake following mechanical stimulation in eRMS (Fig. 8a) and aRMS (Fig. 8b) cells. To determine whether these PANX1 mutants could reduce RMS malignant properties, stable Rh18 (eRMS) and Rh30 (aRMS) cell lines expressing the C66S, C84S, or C265S mutant under the cumate switch system were submitted to 3D spheroid assays. Remarkably, expression of C66S, C84S, and C265S mutants inhibited the formation of eRMS and aRMS spheroids (Fig. 8c) and induced their regression (Fig. 8d) to the same extent as wild-type PANX1, relative to controls. To strengthen our findings, cumate-treated Rh30 cells, representing the most aggressive RMS subtype, expressing the GFP vector or the C265S mutant were injected into mice gastrocnemius muscles and kept under cumate-inducing conditions. While the volume of control tumors increased rapidly, the C265S-expressing aRMS xenografts grew significantly slower (Fig. 8e). Similar to wild-type PANX1, expression of the C265S mutant lead to ~50% reduction in tumor volume at endpoint (Fig. 8f). Collectively, our findings indicate that PANX1 mutants reduce RMS malignant properties in vitro and in vivo despite being deficient in channel function.

## Discussion

Given that RMS arises from skeletal muscle progenitor cells lacking the ability to terminally differentiate, we demonstrate here, using a panel of patient-derived RMS cell lines and RMS tumor specimens, that PANX1 expression is down-regulated in eRMS and aRMS as compared to normal skeletal muscle cells and tissue. Our findings that PANX1 transcript and protein levels in RMS cells were comparable to that of proliferative and undifferentiated skeletal muscle myoblasts suggested that the down-regulation of PANX1 may be involved in the malignant phenotype of RMS. Our complementary data revealed that ectopic expression of PANX1 significantly reduced RMS malignant properties in vitro and in vivo. Importantly, this effect was consistent between eRMS and aRMS despite their heterogeneous background of genetic alterations. Altogether the data presented here constitute the first evidence of PANX1 tumor suppressive functions in human cancer and suggest that increasing PANX1 levels would be a novel therapeutic approach for RMS.

Surprisingly, ectopic PANX1 was incapable of dye uptake when expressed in eRMS (Rh18) and aRMS (Rh30) cells. While Panx1 is thought to form channels only on the plasma membrane^34^, Panx1 channels localized in the ER have been suggested to contribute to ER Ca^2+^ leakage^35^. However, no differences in intracellular Ca^2+^ concentration have been detected in Rh30 and Rh18 cells over-expressing PANX1 compared to control cells when assessed by flow cytometry using the Fura-2 calcium indicator (data not shown). Based on our current knowledge of PANX1 channels, these data suggest that ectopic PANX1 does not form functional channels in RMS. While the possibility remains that non-canonical PANX1 channel activity was not detected or that mechanical stimulation may not be an activator of PANX1 channels in these cells, treatment with CBX and 10PANX failed to reverse or alter the PANX1-mediated reduction in eRMS and aRMS cell proliferation and migration. Furthermore, PANX1 mutations causing deficient channel function did not affect PANX1’s ability to prevent the formation and growth of eRMS and aRMS 3D spheroids and induce their regression. Notably, the C265S PANX1 mutant also had the ability to reduce RMS tumor growth in vivo. Collectively, these complementary approaches suggest that PANX1 tumor suppressive function in RMS involves a mechanism independent of its canonical channel activity.

In skeletal muscle myoblasts, we have shown that PANX1 induces differentiation through a channel-dependent process given that myogenic fusion and differentiation were inhibited by probenecid and CBX^9^. It has been documented that myogenic differentiation involves the release of ATP, activation of purinergic receptors, and rise of intracellular Ca^2+^ levels^36,37^. Furthermore, Panx1 channels mediate the acquisition of myogenic commitment and increased MyoD levels in C_2_C_12_ reserve cells through a mechanism that involves ATP release and purinergic receptor activation^38^. Together, these data suggest that ATP released through PANX1 channels plays a pivotal role in its ability to promote myogenic differentiation. Consequently, the lack of channel activity at the cell surface exhibited by ectopic PANX1 in eRMS and aRMS may explain its inability to trigger their differentiation. Regardless, ectopic PANX1 had a potent inhibitory effect on several RMS malignant properties in vitro, which resulted in a significant reduction of tumor growth in vivo.

While Panx1 over-expression has been shown to reduce murine N2a cell proliferation in vitro, this process was reversed by probenecid^39^. Panx1 has also been shown to reduce the size of rat glioma aggregates. This process could be disrupted by CBX and rescued by exogenous ATP^40^. Conversely, our data suggest that PANX1 lacks its cell surface channel function in eRMS and aRMS cells and that its tumor suppressive effects are mediated through a channel-independent mechanism. Thus, the non-canonical function by which PANX1 triggers inhibition of RMS proliferation, migration, and spheroid growth likely involves a novel molecular mechanism or signaling pathway. While the Panx1/PANX1 interactome has started to be tackled by several researchers and Panx1 protein interacting partners have been identified, the functional relevance of these interactions has either remained unknown or often linked to the modulation of Panx1 channel activity (examples: Panx2, P2X, and P2X7 receptors, α-1D adrenergic receptor)^41^. Of interest, some binding partners are involved in apoptosis, such as caspase-1, caspase-11, and the inhibitor of apoptosis, X-linked inhibitor of apoptosis (XIAP)^42^. In skeletal muscle cells, it has been demonstrated that Panx1 is part of a multiprotein complex that includes the dihydropyridine receptor (DHPR), P2Y2 receptor, as well as caveolin-3, and dystrophin^43^. Interestingly, deletions of the dystrophin gene (*DMD*) were found in RMS specimens and, similar to PANX1, dystrophin over-expression inhibited the invasiveness, migration, and anchorage-independent growth of the human metastatic eRMS cell line RMS176 in vitro^44^.

Taken together, our results show that PANX1 is down-regulated in RMS and that restoration of its levels significantly reduced aRMS and eRMS malignant phenotypes in vitro and in vivo. Our findings also indicate that the tumor suppressive role of PANX1 does not require its canonical channel activity suggesting the existence of a novel, yet to be described, mechanism by which PANX1 functions. In order to understand the molecular mechanism by which PANX1 alleviates malignant properties in RMS, further analyses will be required to reveal the direct PANX1 interactors in these cells together with their downstream signaling pathways, which may also identify other potential new therapeutic targets. The comparison of PANX1 direct interactors in RMS cells to that of skeletal muscle myoblasts may also enable a better understanding of the mechanisms activating and inhibiting PANX1 channels in physiologic and pathologic processes.

## Materials and methods

### Human tissue samples and cell lines

Human samples (seven normal; seven eRMS; six aRMS), collected following informed consent, were obtained from the Department of Pathology and Laboratory Medicine, Children’s Hospital of Eastern Ontario and the Ottawa Hospital, Ottawa, Ontario, Canada, after institutional ethics board approval. Rh18, Rh36, Rh28, Rh30 and Rh41 cell lines were from Dr. P. Houghton (St. Jude Children’s Hospital, Memphis, TN, USA). RD and HEK293T cell lines were from American Type Culture Collection. HSMM were from Lonza and differentiated as previously described^9^. Cells were mycoplasma-negative.

### Plasmid construction, transfections, and stable cell lines generation

*PANX1* cDNA (Origene, Rockville, MD, USA) was subcloned into pCDH-CuO-MCS-EF1-GFP lentiviral vector (System Biosciences, Palo Alto, CA, USA). PANX1 constructs encoding change from cysteine to serine at position 66, 84, or 265 were generated using the QuickChange site-directed mutagenesis kit (Agilent Technologies, Santa Clara, CA, USA) and verified by sequencing.

Transfections used Lipofectamine 2000 Reagent (Thermo Scientific, Waltham, MA, USA). Stable cell lines were generated using the SparQ Cumate Switch Inducible System (System Bioscience). Cells stably expressing CymR repressor (pCDH-EF1-CymR-T2A-Neo lentivector) were transduced with pCDH-CuO-MCS-EF1-GFP, pCDH-CuO-PANX1-EF1-GFP (PANX1), pCDH-CuO-C66S-EF1-GFP (C66S), pCDH-CuO-C84S-EF1-GFP (C84S), or pCDH-CuO-C265S-EF1-GFP (C265S) lentivectors and selected. PANX1 expression was induced with 30 µg/mL cumate.

### RNA extraction, reverse transcription, and quantitative PCR analysis

Total RNA was extracted using RNeasy Mini Kit (Qiagen, Germantown, MD, USA) and reverse transcription was performed using High-Capacity cDNA Reverse Transcription Kit (Thermo Scientific). The synthesized cDNA was used as template for quantitative PCR using iQ™ SYBR^®^ Green Supermix kit (Bio-Rad, Hercules, CA, USA) on Mastercycler ep *realplex* (Eppendorf, Hamburg, Germany) real-time PCR system with specific primers for *PANX1* (forward 5′-CGGAGTACGTGTTCTCGGATT-3′; reverse 5′-CCTGACGCCAGGAGAAAGAA-3′) and glyceraldehyde 3-phosphate dehydrogenase (GAPDH) (forward 5′-CAAGACCTTGGGCTGGGAC-3′, reverse 5′-AGGCTGCGGGCTCAATTTAT-3′). Relative expression was determined using the comparative Ct method.

### Antibodies

Primary antibodies: PANX1 (Sigma-Aldrich, St. Louis, MO, USA; HPA0169300), MHC (R&D Systems, Minneapolis, MN, USA; MAB4470), MyoD (Santa Cruz, Dallas, TX, USA; sc-760), myogenin (Santa Cruz; sc-12732), tubulin (Santa Cruz; sc-8035), GAPDH (Advanced ImmunoChemical, Long Beach, CA, USA; 2RGM2), EGFR (Santa Cruz; sc-03-G), GM130 (Abcam, Cambridge, MA, USA; ab169276), calnexin (Santa Cruz; sc-46669), and BrdU (Thermo Scientific; 03-3900). Secondary antibodies conjugated to Alexa Fluor 405, 488, or 594 (Thermo Scientific; A-31553, A-11001, or A-11012), and Alexa 680-labeled (Thermo Scientific; A21109) or infrared fluorescent-labeled secondary antibodies IRDye 800 (VWR, Radnor, PA, USA; CA610-132-121).

### Immunofluorescence microscopy

Human tissue sections were labeled as previously described^25^. Olympus Fluoview FV-1000 Laser Confocal Microscope image acquisition was performed sequentially with the microscope settings kept constant^25^. Quantification used accompanying analysis software. Positive labeling in skeletal muscle optimized cut-offs for positive labeling^25^. Relative densitometric units were quantified within a constant fixed area.

Cells were immunolabeled as previously described^9^. For comparison of myogenic factor expression, images of 15 random fields (×20 objective) were counted.

### Western blotting

Cell lysates were obtained and analyzed as previously described^9,25^.

### Proliferation assay

Cells were transfected with the empty vector or PANX1. Twenty-four hours post-transfection, cells were incubated with 10 µM BrdU (Sigma-Aldrich) for 3 h (Rh18) or 1 h (Rh30) and processed for immunohistochemistry^9^.

Stable cell lines were incubated with 100 µM CBX (or DMSO) or 200 µM 10PANX (or Scr peptide) for 48 h in the presence of cumate. The assay was performed according to the manufacturer’s protocol (BrdU colorimetric kit, Roche Applied Sciences, Penzberg, Germany).

### Migration assay

A 96-well WoundMaker device (Essen Bioscience, Ann Arbor, MI, USA) created a uniform scratch in cells monolayer. CBX (or DMSO) or 10PANX (or scramble (Scr) peptide) were added at 100 and 200 µM, respectively. Migration was measured by IncuCyte ZOOM Live Cell Imaging System (Essen Bioscience).

### 3D spheroid formation and regression assays

Cumate pre-treated cells were seeded in ultra low adhesion plates. In regression experiments, cumate treatment began only once spheroids had formed (48 h post-seeding). Mean image fluorescence was measured using IncuCyte ZOOM Live Cell Imaging System.

### Viability assay and flow cytometry

Viable cells seeded on agar (BD Biosciences, San Jose, CA, USA)^45^ were counted by Trypan Blue (Thermo Scientific) dye exclusion. For flow cytometry, cells were washed in cold PBS, re-suspended, stained with Pacific Blue-conjugated Annexin V and 7-Aminoactinomycin D (7-AAD) (BD Biosciences), and analyzed using the LSRFortessa X-20 (BD Biosciences) cell analyzer and FACSDiva software^46^.

### Dye uptake assay

Sulforhodamine B dye uptake assay was performed as previously described, and normalized to PANX1 levels^29,30,47^.

### Cell surface biotinylation assay

Cell surface biotinylation assay was performed as previously described^48^.

### Xenograft studies

Experiments were approved by the Animal Care Committee at the University of Ottawa and conducted per the Canadian Council of Animal Care guidelines. Two million cumate-treated cells were injected into the gastrocnemius muscle of 4-week-old to 6-week-old female SCID mice (Charles River Laboratories, Wilmington, MA, USA). PANX1 (three mice for eRMS; 10 for aRMS) or C265S (six mice) mutant expressing cells were injected into the right leg, while the empty vector cells were injected into the left leg^49^. Mice were intraperitoneally injected every 3 days with 50 µL cumate (600 µg/mL) and sacrificed when tumors reached 2000 mm^3^ (*V* = *L* × *W* × *H* × *π*/6 (*L*: length; *W*: width; *H*: height of tumor))^50^.

### Statistics

For all in vitro assays, cells in each group were plated in triplicates, and each experiment was performed three times (*n* = 3). Statistical significance was determined using unpaired two-tailed Student’s *t*-tests and analysis of variance (ANOVA) followed by Tukey’s, Bonferroni, or Dunnett’s post hoc tests. Results are given as mean ± s.d. Results with *P* < 0.05 were considered significant.
